# Metagenomics reveals sediment microbial community response to Deepwater Horizon oil spill

**DOI:** 10.1038/ismej.2013.254

**Published:** 2014-01-23

**Authors:** Olivia U Mason, Nicole M Scott, Antonio Gonzalez, Adam Robbins-Pianka, Jacob Bælum, Jeffrey Kimbrel, Nicholas J Bouskill, Emmanuel Prestat, Sharon Borglin, Dominique C Joyner, Julian L Fortney, Diogo Jurelevicius, William T Stringfellow, Lisa Alvarez-Cohen, Terry C Hazen, Rob Knight, Jack A Gilbert, Janet K Jansson

**Affiliations:** 1 Department of Earth, Ocean and Atmospheric Science, Florida State University, Tallahassee, FL, USA; 2 Earth Sciences Division, Lawrence Berkeley National Laboratory, Berkeley, CA, USA; 3 Institute for Genomic and Systems Biology, Argonne National Laboratory, Lemont, IL, USA; 4 Department of Ecology and Evolution, University of Chicago, Chicago, IL, USA; 5 Biofrontiers Institute, University of Colorado at Boulder, Boulder, CO, USA; 6 Department of Computer Science, University of Colorado at Boulder, Boulder, CO, USA; 7 The Technical University of Denmark, Center for Biological Sequence Analysis, Kongens Lyngby, Denmark; 8 Deconstruction Division, Joint Bioenergy Institute (JBEI), Emeryville, CA, USA; 9 Civil and Environmental Engineering Department, University of Tennessee, Knoxville, TN, USA; 10 Laboratório de Genética Microbiana, Instituto de Microbiologia Paulo de Góes, Universidade Federal do Rio de Janeiro, Rio de Janeiro, Brazil; 11 Ecological Engineering Research Program, School of Engineering & Computer Science, University of the Pacific, Stockton, CA, USA; 12 Civil and Environmental Engineering Department, University of California, Berkeley, CA, USA; 13 Biological Sciences Division, Oak Ridge National Lab, Oak Ridge, TN, USA; 14 Howard Hughes Medical Institute, University of Colorado at Boulder, Boulder, CO, USA; 15 Department of Chemistry and Biochemistry, University of Colorado at Boulder, Boulder, CO, USA; 16 Department of Plant and Microbial Biology, University of California, Berkeley, CA, USA

**Keywords:** DWH oil spill, hydrocarbons, iTag/Metagenomics, microbial community structure, sediments

## Abstract

The Deepwater Horizon (DWH) oil spill in the spring of 2010 resulted in an input of ∼4.1 million barrels of oil to the Gulf of Mexico; >22% of this oil is unaccounted for, with unknown environmental consequences. Here we investigated the impact of oil deposition on microbial communities in surface sediments collected at 64 sites by targeted sequencing of 16S rRNA genes, shotgun metagenomic sequencing of 14 of these samples and mineralization experiments using ^14^C-labeled model substrates. The 16S rRNA gene data indicated that the most heavily oil-impacted sediments were enriched in an uncultured *Gammaproteobacterium* and a *Colwellia* species, both of which were highly similar to sequences in the DWH deep-sea hydrocarbon plume. The primary drivers in structuring the microbial community were nitrogen and hydrocarbons. Annotation of unassembled metagenomic data revealed the most abundant hydrocarbon degradation pathway encoded genes involved in degrading aliphatic and simple aromatics via butane monooxygenase. The activity of key hydrocarbon degradation pathways by sediment microbes was confirmed by determining the mineralization of ^14^C-labeled model substrates in the following order: propylene glycol, dodecane, toluene and phenanthrene. Further, analysis of metagenomic sequence data revealed an increase in abundance of genes involved in denitrification pathways in samples that exceeded the Environmental Protection Agency (EPA)'s benchmarks for polycyclic aromatic hydrocarbons (PAHs) compared with those that did not. Importantly, these data demonstrate that the indigenous sediment microbiota contributed an important ecosystem service for remediation of oil in the Gulf. However, PAHs were more recalcitrant to degradation, and their persistence could have deleterious impacts on the sediment ecosystem.

## Introduction

During the Deepwater Horizon (DWH) oil spill, from April to July 2010, ∼4.1 million barrels of oil were released by the Macondo Well (Zukunft, 2010). A variety of mitigation strategies and natural processes resulted in depletion of ∼78% of the oil (Ramseur, 2010; Kimes *et al.*, 2013). The fate of the remaining 22% is unknown. A deep-sea hydrocarbon plume (at ∼1100 m depth) was observed during the DWH spill and was the focus of considerable attention, especially regarding the potential for microbial degradation of hydrocarbon contaminants. Shortly after the spill, uncultured *Oceanospirillales* dominated the deep-sea plume (Hazen *et al.*, 2010; Mason *et al.*, 2012; Redmond and Valentine, 2012). A combination of metagenomics, metatranscriptomics and single-cell genome sequencing revealed that representatives of this dominant clade were active and capable of degrading cycloalkanes (Mason *et al.*, 2012). Over time, *Oceanospirillales* was supplanted by *Colwellia* and *Cycloclasticus* (Valentine *et al.*, 2010). Redmond and Valentine (2012) used stable isotope probing in microcosm experiments and reported that *Colwellia* was likely active in ethane, propane and benzene oxidation in the deep-sea plume. Later, after the spill ceased, methylotrophs, some of which are known methane oxidizers, dominated the region of the plume (Kessler *et al.*, 2011).

This succession of microbial community members was replicated in laboratory microcosm studies during which large flocs, also known as ‘marine snow', comprised largely of *Colwellia*, extracellular polymeric substances and oil, accumulated in the water (Bælum *et al.*, 2012a). Similarly, during the spill in the Gulf of Mexico, an unusually large marine snow event occurred in surface waters (Passow *et al.*, 2012). One hypothesis to explain this event is that hydrocarbon-degrading bacteria produced oil-emulsifying extracellular polymeric substances, resulting in large sticky aggregates/snow (Passow *et al.*, 2012). Within one month, however, the marine snow was no longer observed in the Gulf of Mexico (Passow *et al.*, 2012), suggesting that it might have been degraded. Alternatively, the flocs and remaining contaminants may have settled to the sea floor where their fate remains unknown, in what has been termed the ‘dirty blizzard' phenomenon (Schrope, 2013). These sinking aggregates could contain fossil carbon and perhaps unassimilated hydrocarbons, a fraction of which could have reached the sediments (Passow *et al.*, 2012). This, in addition to oil that was directly deposited onto sediment surfaces during the spill, may partly explain the fate of some of the 1.1 million barrels of oil that were unaccounted for as of August 2010 (Ramseur, 2010). The ‘dirty bathtub' mechanism, in which deep-sea sediments were contaminated by impacted water as it circulated throughout the Gulf of Mexico basin (Schrope, 2013), could also have deposited hydrocarbons on the seafloor. In fact, by August 2010, deep-sea sediments had been contaminated by the oil spill, with several samples collected within 3 km of the wellhead exceeding the Environmental Protection Agency (EPA)'s aquatic life benchmarks for polycyclic aromatic hydrocarbons (PAHs), here abbreviated as EPA-BM (Zukunft, 2010).

Here we sought to characterize the impact of the DWH spill on the microbial community in the deep sea at the sediment seawater interface (surface sediments). To address these aims, we used a combination of deep-sequencing approaches, bioinformatics and activity measurements to analyze deep-sea sediments that were collected between September and October 2010, after the wellhead was capped in July 2010. We also compared the microbial community and metabolic processes in the contaminated deep-sea sediments with those found previously in the water column hydrocarbon plume.

## Materials and methods

### Sample collection and processing

Sediment cores were collected as part of the OSAT R/V Gyre cruise from 16 September to 20 October 2010. Sediment samples were obtained at an average water depth of ∼1500 m below sea level. Sample metadata and chemistry are provided in Supplementary Table 1. Samples that were collected using a Mega Corer were capped and frozen at −80 °C. Frozen sediment cores were sectioned (0–1 cm) using a 12″ double-bevel sliding compound miter saw (Dewalt DW 716, Baltimore, MD, USA) and Fast-Cut diamond-grit-edge saw blades for multiple materials (McMaster-Carr, Sante Fe Springs, CA, USA). Saw blades were power-washed (model CM-1400-OMEH, Mi-T-M Corporation, Peosta, IA, USA), soaked in 70% ethanol for at least 5 min and then rinsed in sterile, distilled water between cores. Fluorescent beads were used to check for carry-over between cuts. A bead suspension was sprayed onto the saw blades, blades were washed or not washed, and then sections were examined for the presence of beads. Clean aluminum foil was used to cover work surfaces and was disposed of after completing core cutting and before cutting the next core. The surface 0–1 cm sections of each core were selected for all of the subsequent analyses. Core sections were chiseled into wedges, and scraped using sterile scalpels to remove the outer layers of all exposed sides. The sample location map and subsequent plots of hydrocarbon and nitrogen concentrations were made with Ocean Data View (Schlitzer, 2013).

### Hydrocarbons

To determine hydrocarbon concentrations in sediment samples, 50–1000 μl of chloroform was added to neutral lipid extracts (depending on anticipated oil concentration); samples were vortexed and then sonicated for 30 s. Neutral lipid extracts were analyzed by GC/FID and peaks identified by GC/MS (Agilent, Santa Clara, CA, USA). Quantification was accomplished by comparing with a known hexadecane standard curve. BP staff determined whether samples exceeded the EPA-BM according to http://www.epa.gov/bpspill/water/explanation-of-pah-benchmark-calculations-20100622.pdf on duplicate core samples. In addition, BP determined PAH and organic carbon concentrations and did the calculations. Briefly, as described by the EPA, PAH concentrations (dry weight) were normalized by dividing by the fraction of organic carbon. Next, normalized values were divided by their potency divisors.

### Nutrients

Frozen sediment samples were weighed and measured (length, height and width). Thawed samples were centrifuged at 3000 r.p.m. for 5 min. The supernatant was removed using a pipette and filtered to remove sediment particles. Approximately 5 ml of porewater was used for each analysis. Interstitial water was analyzed by methods outlined in Standard methods for the analysis of water and wastewater (APHA, AWWA, WEF, 2005). Briefly, interstitial water samples used for dissolved ammonia nitrogen (NH_3_-N and NH_4_-N, a.k.a., total ammonium-N or TAN), dissolved nitrate nitrogen (NO_3_-N) and dissolved phosphate (PO_4_-P) were filtered through 45 μm GHP membrane filters (Pall Life, Port Washington, NY, USA) before analysis. Dissolved inorganic nitrogen components (NH_3_-N and NO_3_-N) were quantified using the TL-2800 ammonia analyzer (Timberline Instruments, Boulder, CO, USA). Ortho-phosphate (PO_4_-P) was quantified by the ascorbic acid method adapted from SM 4500-P-E in Standard methods for the analysis of water and wastewater (APHA, AWWA, WEF, 2005). Unfiltered samples were subjected to a persulfate digestion before analysis of total phosphorus (TP). The digested samples were quantified by the ascorbic acid method adapted from SM 4500-P-E (APHA, AWWA, WEF, 2005).

Total carbon, nitrogen and sulfur (total C, N and S) were measured in sediments using a Thermo Scientific Flash2000 Organic Elemental Analyzer (Waltham, MA, USA). Before total C, N and S analysis, sediment samples were dried at 105 °C under vacuum to constant weight and then ground to a fine powder using a Hard Tissue Homogenizer (VWR International, West Chester, PA, USA). Total N includes NH_3_/NH_4_-N, NO_3_/NO_2_-N and organic N in the whole sediment sample after sample drying. Approximately 10 mg of powdered sample was combusted within the instrument at 900 °C, and the resulting gases were separated using a chromatography column and measured with a thermal conductivity detector referenced to helium carrier gas.

### DNA extraction

For DNA extractions from sediment samples, 0.25 g of sediment was extracted in quadruplicate using the Earth Microbiome Project DNA extraction protocol (http://www.earthmicrobiome.org/emp-standard-protocols/dna-extraction-protocol/). Briefly, this protocol uses a modified PowerSoil-htp 96 well DNA isolation kit (MoBio, Carlsbad, CA, USA) with an epMotion 5075 Vac robotic platform (Eppendorf, Hauppauge, NY, USA). Quadruplicate samples were pooled after extraction.

### 16S rRNA gene sequencing and Analysis

16S rRNA genes were amplified from the DNA extracts in triplicate using archaeal and bacterial primers 515F and 806R, which targets the V4 region of *E. coli* in accordance with the protocol described by Caporaso *et al.* (2011, 2012) and used by the Earth Microbiome Project (http://www.earthmicrobiome.org/emp-standard-protocols/16s/). Samples were analyzed using the QIIME version 1.5.0-dev (Caporaso *et al.*, 2010a) pipeline. Raw sequences were demultiplexed and then quality-filtered using the default parameters in QIIME. Sequences were then clustered into operational taxonomic units (OTUs, 97% similarity) with UCLUST (Edgar, 2010) using the open reference clustering protocol (http://qiime.org/tutorials/open_reference_illumina_processing.html). The October 2012 release of Greengenes (McDonald *et al.*, 2012) was used. The resulting representative sequence set were aligned using PyNAST (Caporaso *et al.*, 2010b) and given a taxonomic classification using RDP (Wang *et al.*, 2007) retrained with the Greengenes release. The resulting OTU table was filtered to keep only OTUs that had at least 10 sequences, and then rarefied at 122 804 sequences per sample (the minimum number of remaining sequences in any of the samples). The QIIME generated, rarefied, OTU abundances in the 64 different samples were then analyzed using non-metric multidimensional scaling in R with the Vegan package. *P*-values are derived from 999 permutations of the data.

### Metagenomic library preparation, sequencing and quality assessment

Metagenomic shotgun sequencing libraries were prepared and sequenced at Argonne National Laboratory. For each sample, 1 μg of genomic DNA was used with Illumina's TruSeq for library preparation. Libraries were sequenced using the Illumina HiSeq 2 × 100-bp paired-end technology. Each sample was run in a single lane. This approach generated 1 Tb of sequence data for all 14 samples. Sequences that had ⩾5% bp with ⩽10 phred scores were filtered out before bioinformatics analyses.

### Metagenomic sequence annotation and bioinformatics

Raw, unassembled reads were annotated in MG-RAST (Meyer *et al.*, 2008) using Hierarchical Classification subsystems with a maximum e-value cutoff of 10^−5^, a minimum percent identity cutoff of 60% and a minimum alignment length cutoff of 15. MG-RAST annotation data were analyzed by network analysis. Networks were generated using QIIME 1.6.0-dev and visualized using Cytoscape 2.8.3. Edges connecting a sample to a function were omitted if the function was observed fewer than 20 times in that sample. The network layout was generated using Cytoscape's edge-weight spring-embedded algorithm, where the edge weights corresponded to abundance. These functional annotations were also evaluated using STAMP (Parks and Beiko, 2010) after the data were normalized. Specifically, samples that exceeded the EPA-BM were compared with those that did not exceed the EPA-BM by using a two-sided Welch's *t*-test, with confidence intervals of 0.95 with the following filter: difference between proportions, effect size <1.00. These raw reads were also annotated by comparing them with a database of proteins involved in hydrocarbon degradation using USEARCH v.6. (Edgar, 2010). For each sequence, the result with the highest bit score was selected with a bit score cutoff of ⩾40. Before statistical analysis data were normalized. These normalized data were analyzed using STAMP as previously described. Further, the most abundant genes involved in hydrocarbon degradation in sediment samples were compared with similar metagenomic data from the deep-sea plume that were presented by Mason *et al.* (2012).

### PRMT

Predictive relative metabolic turnover (PRMT) predicts the relative metabolic activity as a function of microbial metabolic community potential (Larsen *et al.*, 2011). PRMT is a comparison between two sample (or a sample and a reference sample) sets of genetic sequence annotations attributed to enzyme commission numbers transformed by a weighted matrix of possible metabolic reactions (the environmental transformation matrix) to determine the metabolic turnover of the community. Thus, PRMT evaluates the relative relationships of enzymes across the set of potential pathways in the community. The outcome of PRMT analysis is a set of metabolites and their associated scores. For this analysis we used the average of all samples as the reference. Positive scores are consumption of a particular metabolite, and negative scores are accumulation of a particular metabolite. Raw, unassembled reads were annotated in MG-RAST using SEED, with a maximum e-value of 1 × 10^−3^, a minimum identity of 50% and a minimum identity cutoff of 15. Samples were quantile normalized and log 2-transformed before analysis. For this analysis we used the average of all samples as the reference. The environmental transformation matrix data were collected from the KEGG database (Ogata *et al.*, 1999) as of September 2010. Metabolite scores were annotated back to function and compared between samples that exceeded EPA-BM and those that did not exceed EPA-BM. For sample comparisons, Wilcoxon-signed rank tests were used, and 1000 permutation tests were used to determine *P*-values. The PRMT analysis was completed in R and perl (scripts available upon request).

### ^14^C experiments

Approximately 400 g of sediment was pooled from the upper 1–2 cm from cores BP233, BP237, BP241, BP249, BP253, BP261 and BP273. Material from the 0–1-cm sediment interval was not available for this assay. The sediment was thawed, homogenized and stored in a 1-l airtight redcap flask at 4 °C for 8 days before initiating experiments. Microcosms were set up in triplicate in 30 ml glass flasks with teflon-coated airtight stoppers (Bellco, Vineland, NJ, USA). In all, 5 g of sediment (wet weight) was placed inside each flask. To ensure water-saturated conditions throughout the incubation period, 2 ml of the seawater obtained from the cores was added. Radioactive substrates were added as traces of 50 000 DPM U-ring ^14^C-labeled toluene or phenanthrene (radiochemical purity of >97% Sigma-Aldrich, St Louis, MO, USA) or 1-C ^14^C-labeled dodecane (radiochemical purity of >98% Sigma-Aldrich) and mixed with unlabeled dodecane, toluene or phenanthrene (chemical purity of >98% Sigma-Aldrich). Carbon substrates were 10 mg kg^−1^ sediment. A CO_2_ trap consisting of a 0.8-ml glass vial containing 300 μl of 1 M NaOH was placed inside the glass flask and mineralization was measured as ^14^CO_2_ trapped in NaOH, as described previously (Bælum *et al.*, 2012b). The microcosms were incubated in the dark at 5 °C throughout the 92-day incubation period. Up to day 40, the NaOH trap was replaced every 2–4 days, whereas after day 40 it was replaced every 10–20 days. Two types of control microcosms were initiated. The first control type was sterilized by autoclaving three times and adding 100 mg kg^−1^ sodium azide. The second type contained no carbon. The no-carbon controls were used to measure background radiation, and sterilized microcosms served as control for trapping of evaporated ^14^C-compounds. The results were corrected accordingly.

## Results and discussion

**Figure 1:**
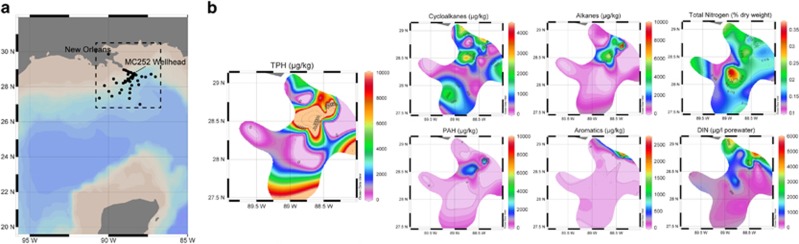
(**a**) Map of 64 sample sites and (**b**) Their corresponding TPH, alkane, cycloalkane, PAH, total nitrogen and DIN concentrations. Note that the data shown represent discontinuous samples. For the sake of comparison, an effort was made to scale down to the lowest hydrocarbon concentration (cycloalkane); the maximum TPH concentration was 65 643 μg kg^−1^, 29 338 μg kg^−1^ for alkanes and 9075 μg kg^−1^ for PAH. Total nitrogen and DIN were not scaled down.

A total of 64 sediment cores were collected between September and October 2010 (4–5 months following the spill) in several directions around the wellhead at distances ranging from 0.3 to 256 km (Figure 1, Supplementary Table 1), from which the surface 0- to 1-cm sediments were analyzed here. Samples collected nearest the wellhead (19 samples from within 5 km) had the highest concentration of total petroleum hydrocarbons (TPH; average of 19 258 μg kg^−1^) (Figure 1, Supplementary Table 1) and were the only samples that exceeded the EPA-BM (Supplementary Table 1). The more distant samples were relatively uncontaminated, the lowest TPH concentration being 18 μg kg^−1^ (Figure 1 and Supplementary Table 1). It should be noted that the data shown in Figure 1 do not assume a continuous distribution of hydrocarbon concentrations between the sampling points.

**Figure 2:**
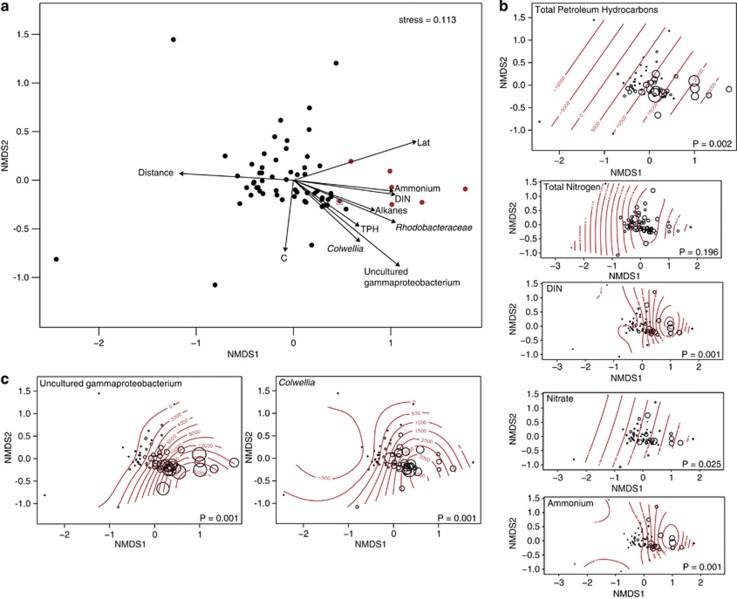
Non-metric multidimensional scaling ordination of 16S rRNA gene iTag sequence data. (**a**) The main ordination shows sample similarity and the correlations between environmental variables and ordination axes. The three most abundant OTUs in the contaminated samples (uncultured *Gammaproteobacterium*, *Colwellia* and *Rhodobacteraceae*) are represented by arrows. Sample BP366 is indicated by a blue square. Samples that exceeded the EPA's aquatic benchmark for PAHs are denoted by red circles. (**b**) For this same ordination, the concentrations of TPH, total nitrogen, DIN, nitrate and ammonium are indicated by bubble size and contour lines. (**c**) Rarified abundance of an uncultured *Gammaproteobacterium* OTU and a *Colwellia* OTU are shown for the same ordination, with bubble size and contour lines indicating abundance. *P*-values indicate the significance for the variable shown and are based on 999 permutations.

The microbial community (beta diversity) was structured according to gradients in (DIN; NH_3_ and NO_3_) and TPH concentrations, with samples that had higher DIN concentrations and exceeded EPA-BM forming a distinct cluster (Figure 2), except for sample BP366, which grouped closer to samples that did not exceed EPA-BM (Figure 2). Sample BP366 had a relatively low PAH concentration (208 μg kg^−1^) and had no other detectable aromatic compounds, which may explain the observed discrepancies in clustering between exceeded and nonexceeded samples (Figure 2). The microbial data were therefore useful for recognizing that this sample likely represented a midpoint sample on the TPH/PAH concentration continuum, and we took this into account in several subsequent analyses discussed below.

The contaminated sediments showed a significant increase in the relative abundance of Proteobacteria (Supplementary Figure 1), especially of an uncultured *Gammaproteobacterium* represented by a single OTU; (97% similarity) (Greengenes OTU ID 248394), that comprised up to 18% of the microbial community in highly contaminated samples, but <0.01% in the other samples (Figure 2). The OTU was 99–100% similar, over the partial V4 region sequenced, to several sequences previously reported from the DWH deep-sea plume (Kessler *et al.*, 2011; Redmond and Valentine, 2012), surface sediments from Guaymas Basin hydrothermal vents (Teske *et al.*, 2007) and hydrocarbon seep sediments, described as ‘oily sediment, hydrate' in the Gulf of Mexico (Kleindienst *et al.*, 2012).

A *Colwellia* taxon (OTU ID 97018) also had a similar distribution pattern to the *Gammaproteobacterium* discussed above, ranging in relative abundance from 6% in highly contaminated samples to undetectable in some samples with low TPH concentrations. This OTU had 100% sequence similarity to several other previously described *Colwellia* sequences, including a *Colwellia* clone from the DWH deep-sea plume (Valentine *et al.*, 2010), *Colwellia psychrerythraea* strain 34H (Methé *et al.*, 2005), and a *Colwellia* strain previously shown by stable isotope probing to incorporate methane, propane, ethane and benzene (Redmond and Valentine, 2012). However, in the SIP study, crossfeeding could not be ruled out. This OTU was also 94% similar to *Colwellia* RC25, which was shown to degrade MC252 oil that was collected during the DWH spill (Bælum *et al.*, 2012a).

An uncultured *Alphaproteobacterium* (OTU ID 567191) in the *Rhodobacteraceae* family was also highly abundant (13%) in one of the samples that exceeded the EPA-BM (BP315), whereas it was much less abundant (3% to negligible) in other samples (Supplementary Figure 2). This OTU was closely related (100% similar) to a microorganism observed in the aforementioned SIP experiments that appeared to incorporate ethane and propane (Redmond and Valentine, 2012). It was also similar (88%) to a sequence from another SIP experiment conducted with [^13^C]-naphthalene (Gutierrez *et al.*, 2011), suggesting that it may have been involved in gaseous hydrocarbon and PAH degradation, but this remains to be confirmed.

To date, few other studies of deep surface sediments during or after the DWH spill are available. Recently, Kimes *et al.* (2013) evaluated deeper layers (1.5–3 cm below seafloor) of three sediment cores from the Gulf of Mexico, two of which we included in this study (BP315 and BP278). They suggested that the oil spill enriched anaerobic hydrocarbon-degrading *Deltaproteobacteria* in the deeper sediment layers. In the present study, the overlying surface sediment layers were enriched with uncultured *Gammaproteobacteria* similar to those previously observed in the deep-sea hydrocarbon plume. In particular, the increase in relative abundance of a *Colwellia*, closely related to *C. psychrerythraea* strain 34H, a facultative aerobe, with the denitrification pathway encoded in its genome (Methé *et al.*, 2005), suggested that facultative aerobes were present in the surface sediments. However, the dominant anaerobic hydrocarbon-degrading *Deltaproteobacteria* described by Kimes *et al.* (2013) were not abundant in our samples. These findings suggest that aerobic and anaerobic processes were being carried out in the surface sediments but that the dominant microbial players differed with depth and that this may be related to differences in redox. In addition to Kimes *et al.* (2013) the only other published study to our knowledge was by Liu and Liu (2013) who analyzed two sediment samples obtained 2–6 km away from the wellhead 1 year after the spill began and found that methanotrophs, *Pseudomonas*, *Vibrio*, *Flavobacteria* and *Acidobacteria* were dominant in the 0–2-cm surface sediments. These groups were, however, not prevalent in our samples. The difference between these two studies could be due to sampling at different sites that were more distant from the DWH wellhead compared with the closer samples that exceeded EPA-BM that we included here. We sampled 5 months after the spill began (2 months after cessation), whereas Liu and Liu (2013) sampled more than 1 year after the spill began, and thus differences could also be due to successional dynamics of the community with a shift toward a methanotroph-dominated system.

Correlation of the environmental data to the microbial community compositions in the samples revealed that both hydrocarbon and inorganic nitrogen concentrations were important in determining how the microbial communities were structured (Figure 2). Thus, hydrocarbon and nitrogen cycling pathways were explored in greater detail using shotgun metagenome sequencing of a subset of 14 sediment samples ranging from those that were highly contaminated to those with nearly undetectable levels of hydrocarbons. The samples selected for metagenome sequences were characterized *a priori* as either exceeding or not exceeding the EPA-BM (seven in each category). Approximately 1 Tb of paired-end sequence data were obtained, thus representing one of the deepest metagenome sequencing projects available to date. The raw reads were annotated using MG-RAST and by comparison to a custom database containing genes involved in hydrocarbon degradation.

Annotation of metagenomic fragments against the MG-RAST (Meyer *et al.*, 2008) M5nr database with hierarchical classification revealed 2860 total functional annotations, with 184 unique functions ascribed to samples that did not exceed EPA-BM, compared with 660 in samples that did exceed EPA-BM (Supplementary Figure 3). Genes involved in sulfur metabolism, nucleosides and nucleotides, secondary metabolism, membrane transport, cell wall and capsule, protein metabolism and metabolism of aromatic compounds were detected at significantly higher levels in samples that exceeded EPA-BM, compared with those that did not (Supplementary Figure 4).

The metagenome data revealed that deposition of hydrocarbons from the DWH spill resulted in an increase in the functional repertoire of surface sediment microorganisms, particularly those responsible for degradation of alkanes and aromatic hydrocarbons. Functional gene categories involved in metabolism of aromatic compounds were significantly more abundant in samples that exceeded EPA-BM, all seven of which clustered together (Supplementary Figure 5). Genes involved in the metabolism of central aromatic intermediates, peripheral pathways for catabolism of aromatics and anaerobic degradation of aromatics were more abundant than those that did not exceed the EPA-BM (Supplementary Figure 5), except for sample BP366, which grouped with samples that did not exceed (Supplementary Figures 6 and 7). Network analysis of these annotations also supported this conclusion (Supplementary Figure 3).

**Figure 3:**
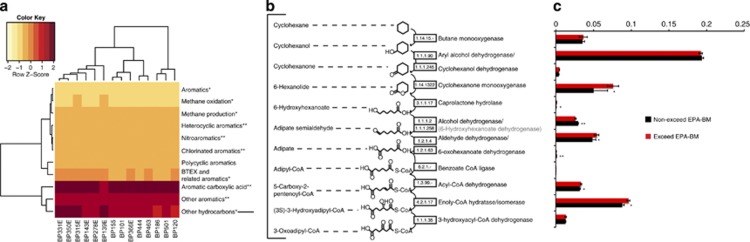
Metagenomic data annotated by comparing raw reads with a database of genes involved in hydrocarbon degradation. (**a**) The heatmap shows abundance of genes involved in degradation of a particular hydrocarbon. (**b**) The dominant hydrocarbon degradation pathway is shown, along with (**c**) A statistical comparison of these gene abundances in samples that exceeded the EPA-BM and those that did not. *Genes that were statistically significantly different and more abundant in samples that exceeded EPA-BM ; **Those that were more abundant in the nonexceed samples.

To further resolve differences in the potential for degrading aromatics (and other hydrocarbons), specific pathways involved in hydrocarbon degradation in the sediment samples were characterized by comparing raw reads with a custom database of genes involved in hydrocarbon degradation that was previously used for analysis of functional genes in the deep-sea hydrocarbon plume (Mason *et al.*, 2012). The results confirmed that there were statistically significant differences in potential degradation of some aromatics when comparing exceed with nonexceed samples (Figure 3). For example, in samples that exceeded the EPA-BM, genes involved in degradation of BTEX compounds were more abundant. However, there was no difference in the abundance of genes involved in PAH degradation (Figure 3). Some of the most abundant genes retrieved in this analysis were those that fall under ‘other hydrocarbons' (Figure 3). Specifically, the most abundant genes were aryl alcohol dehydrogenase, enoly-CoA hydratase/isomerase, cyclohexane monooxygenase, aldehyde dehydrogenase and butane monooxygenase (Figure 3). From these data, a complete pathway for cyclohexane degradation was recovered (Figure 3). This pathway is similar to that described for *Brachymonas petroleovorans* CHX that was isolated from a wastewater plant of a petroleum refinery (Rouvière and Chen, 2003). *B. petroleovorans* is able to grow on a wide range of hydrocarbons, including cyclic alkanes, linear alkanes (C_5_–C_10_), aromatics (toluene, benzyl alcohol and m-xylene) and gasoline (Rouvière and Chen, 2003). In *B. petroleovorans*, cyclohexane is first oxidized by a butane monooxygenese homolog closely related to the soluble butane monooxygenase of *Pseudomonas butanovora* and methane monooxygenases of methanotrophs (Brzostowicz *et al.*, 2005). The cyclohexanol intermediate (Stirling *et al.*, 1977; Trower *et al.*, 1985) is then oxidized by cyclohexanone monooxygenase to adipic acid (Brzostowicz *et al.*, 2005). We reconstructed a similar and complete cyclohexane pathway from the sediment metagenomic data (Figure 3), suggesting that the surface sediment community had the specific capacity for cyclohexane degradation, in addition to more general pathways for degrading other aliphatics and simple aromatics.

**Figure 4:**
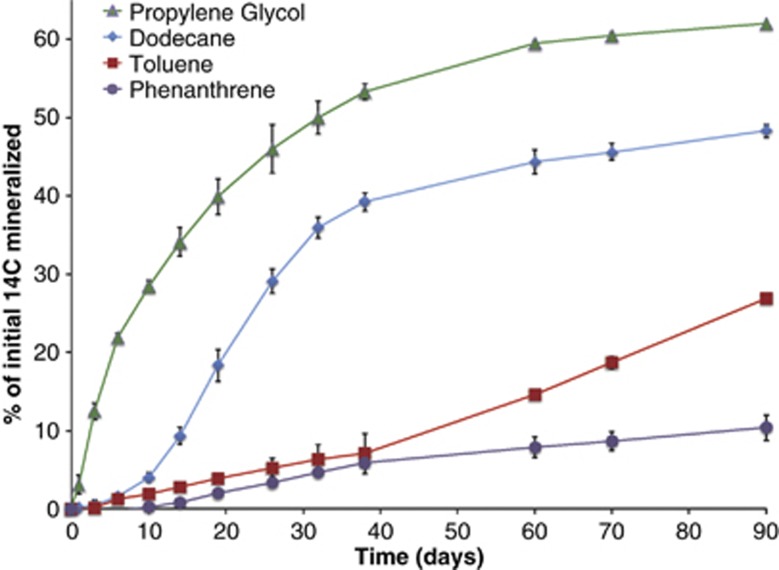
Mineralization data for sediments incubated with ^14^C-labeled propylene glycol as a model component of the dispersant (COREXIT) and ^14^C model compounds found in oil.

To experimentally validate the ability of the sediment community to degrade specific hydrocarbon compounds, we conducted microcosm studies with ^14^C-labeled model compounds and followed their mineralization rates over 90 days of incubation (Figure 4). The model compounds chosen were polyethylene glycol (a component in Corexit, the dispersant used during the DWH spill), dodecane (model linear alkane), toluene (model simple aromatic) and phenanthrene (model PAH). Of these, polyethylene glycol was the most readily degraded with a negligible lag phase (Figure 4). This contrasts to the slower degradation observed by indigenous microbial communities in the water column (Bælum *et al.*, 2012a), suggesting that the sediment microbiota has a greater affinity for some of the compounds in the dispersant. Dodecane was also readily degraded, with an average of 48% mineralized after 90 days, whereas toluene was degraded more slowly, with an average of 27% mineralized by 90 days (Figure 4). Phenanthrene, a three-ring PAH, was initially mineralized at the same slow rate as toluene; however, by day 40, the rate of toluene mineralization increased, and phenanthrene reached a maximum average of ∼10% mineralization over the course of the experiment (Figure 4). These data confirmed our hypothesis derived from the metagenomic data that alkanes and simple aromatics could be degraded by native sediment microbes. Genes involved in PAH degradation were neither predominant in the metagenomic data set, nor was there a significant difference between the samples that exceeded the EPA-BM and those that did not, consistent with the slow rate of PAH degradation that we observed in ^14^C-mineralization experiments. Taken together, the surface sediment microbial community most heavily impacted by the oil spill did not appear to be degrading PAHs. Our findings were confirmed by a recent report that recalcitrant PAHs persisted in sediments that were impacted by the DWH oil spill with negative impacts on meiofauna diversity (Montagna *et al.*, 2013).

Other nonhydrocarbon-degrading pathways (for example, nitrogen cycling) were also significantly altered in samples that exceeded the EPA-BM; for example, the concentration of ammonium was higher in these samples than in those that did not exceed EPA-BM (Figure 2 and Supplementary Table 1). Thus, the deposition of hydrocarbons from the DWH oil spill on the sediment surface likely perturbed the nitrogen cycle. Given that nitrogen metabolism pathways are a network of interactions, PRMT (Larsen *et al.*, 2011) scores were used to determine which pathways in the nitrogen cycle demonstrated significant shifts in predicted metabolite turnover between the samples that exceeded the EPA-BM and those that did not. The PRMT analysis suggested that the capacity of the microbial community to degrade or consume key metabolites in nitrogen cycling pathways was affected by hydrocarbon contamination. Specifically, PRMT predicted an accumulation of nitrogen, nitrite and nitric oxide, as well as the increased potential consumption of nitrate and ammonia (Supplementary Figure 8). This suggests that: (1) nitrogen fixation, together with mineralization, could have resulted in the higher ammonium concentrations in the EPA-BM exceed samples; (2) nitrate may have been consumed via denitrification.

**Figure 5:**
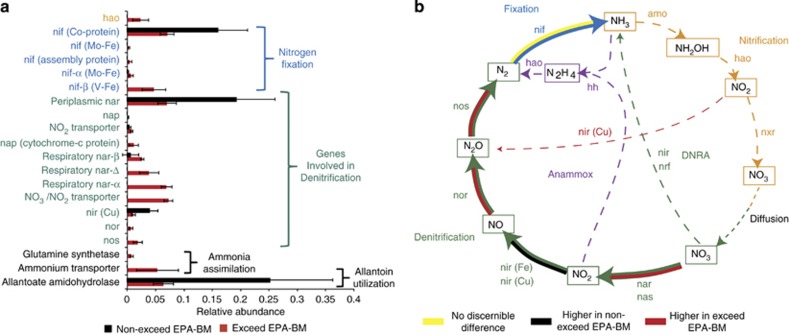
Genes involved in nitrogen cycling. (**a**) Only those genes that were statistically different when comparing samples that exceed EPA-BM with those that did not. (**b**) Nitrogen cycle displaying statistically significant differences when comparing samples that exceed EPA-BM with those that did not. Solid lines depict strong support for a pathway (on the basis of the metagenomic data sequence abundance), whereas dashed lines indicate little to no support for a pathway. Abbreviations: amo, ammonia monooxygenase; hao, hydroxylamine oxidoreductase; hh, hydrazine hydrolase; nar, nitrate reductase (dissimilartory); nas, nitrate reductase (assimilatory); nif, nitrogenase (various types); nir (Fe/Cu), nitrite reductase (Fe/Cu containing); nor, nitric oxide reductase; nos, nitrous oxide reductase; nrf, nitrate reductase (associated with nap); nxr, nitrite oxidoreductase.

On the basis of these predictions, analysis of the specific relative abundances of several genes catalyzing different redox reactions in the nitrogen cycle helped generate a description of the dominant N-cycling processes in these sediments (Figure 5). As predicted by PRMT, nitrogen fixation appears to be the primary source of ammonia input into these sediments, and variants of the nitrogenase gene were found in both samples that exceeded EPA-BM and those that did not (Figure 5). There was little evidence for a change in the relative abundance of genes that catalyze nitrification (NH_3_→NO_3_) (Figure 5). However, nitrification is a strictly aerobic process (Ward, 2008). The surface sediments analyzed in our study likely experienced a sharp decline in redox from the surface to 1 cm depth. This could explain why both aerobic and anaerobic processes were predicted in the metagenome data. Furthermore, nitrification is inhibited under conditions of high organic matter loading owing to competition between nitrifiers and heterotrophs for ammonia (Austin *et al.*, 2006); therefore, it is likely that the potential for nitrification would be underrepresented in the hydrocarbon-contaminated samples.

The majority of annotated genes in the metagenome represented pathways in the denitrification leg of the nitrogen cycle (NO_3_→NO_2_→NO→N_2_O→N_2_), suggesting that denitrification was the dominant N-loss process. The genome of *C. psychrerythraea* strain 34H (discussed above) encodes the denitrification pathway (Methé *et al.*, 2005). Given the concomitant increase in relative abundance of a *Colwellia*, closely related to *C. psychrerythraea* strain 34H, and genes involved in denitrifictation, we directly compared raw metagenome reads with *C. psychrerythraea* using MG-RAST. There was a high sequence similarity between genes annotated as involved in nitrogen cycling, and in particular denitrification in *C. psychrerythraea* in comparison with metagenomic sequences (average similarity was 87% of all nitrogen cycling genes). Of note was the increase in similarity of *C. psychrerythraea*'s nitrate reductase proteins, *Nap*D, *Nap*E and nitrate reductase catalytic activity in samples that exceeded the EPA-BM (average 88% similar) compared with those that did not (average 73% similar). Thus, denitrification, specifically nitrate reduction, could be ascribed to sediment microbes closely related to *C. psychrerythraea*, particularly in samples that exceeded EPA-BM.

One hypothesis to explain these results is that the oil deposition, and possibly biomass deposition together with flocs that formed in the water column, resulted in sufficient carbon to drive respiration and lower the effective oxygen concentration on the sediment surfaces to the point that nitrate was reduced through denitrification. As previously discussed, the analysis of microbial community structure revealed that NH_3_ and NO_3_ were highly correlated with axis 1, and thus the differences in community structure along this axis could be directly related to gradients in nitrogen species concentrations, which agrees with the PRMT predictions.

Comparison of the metagenomes derived from the sediments with those previously obtained from the deep-sea plume in the water column revealed several differences in microbial community compositions and hydrocarbon degradation potential. For example, dominant microbes in the deep-sea hydrocarbon plume included an uncultured *Oceanospirillales* (Hazen *et al.*, 2010; Mason *et al.*, 2012), *Colwellia* (Valentine *et al.*, 2010; Redmond and Valentine 2012), *Cycloclasticus* (Valentine *et al.*, 2010) and methylotrophs, some of which are capable of methane oxidation (Kessler *et al.*, 2011). As described above, the dominant microbial populations in the deep-sea surface sediments, particularly the more heavily impacted samples, were markedly different from what was reported in the water column, except for *Colwellia*, which was abundant in certain samples in both environments. *Colwellia* has been shown to degrade both gaseous alkanes and aromatics (Valentine *et al.*, 2010; Redmond and Valentine 2012) and, more recently, PAHs (Gutierrez *et al.*, 2013). The broad substrate utilization capability of *Colwellia* may, in part, explain why it was abundant in both sediments and the water column, and points to the important role this genus played in degrading hydrocarbons introduced by the oil spill. It is possible that flocs rich in *Colwellia*, as shown in Bælum *et al.* (2012a, 2012b), formed in the deep-sea plume and were deposited on the surface sediments, similar to the dirty blizzard mechanism for surface waters (Schrope, 2013). The codeposition of hydrocarbons with the *Colwellia* could facilitate their catabolism on the surface of deep sediments.

In contrast to the high relative abundance of *Oceanospirillales* in the plume, this order was not as abundant in surface sediments, with an average relative abundance in all samples of 0.8% (the highest was 2%). Although *Oceanospirillales* has repeatedly eluded cultivation efforts (Mason *et al.*, 2012; Gutierrez *et al.*, 2013), sequencing revealed the dominant pathway encoded in the core single-cell genome for this taxon was cyclohexane degradation. In the sediments genes coding for cyclohexane degradation were abundant, but by a different pathway, similar to that described for *B. petroleovorans* (Rouvière and Chen, 2003), than what was reported in the deep-sea plume. In sediments cyclohexane and additional pathways for alkane and aromatic degradation were enriched.

The clear clustering of sediment samples compared with water column samples (Supplementary Figure 9) provides strong evidence that the dominant hydrocarbon degradation pathways were disparate, with aliphatic degradation dominating in the plume metagenomes (Mason *et al.*, 2012), compared with both aliphatic and aromatic processes in the sediments. One commonality was the lack of transcripts involved in PAH degradation in the plume (Mason *et al.*, 2012 and Rivers *et al.*, 2013) and the low percentage mineralization of PAHs in the sediment surface, as reported herein, which suggests that PAHs may have accumulated and persisted. This substantiates the need to characterize the sediment microbial community hydrocarbon degradation capacity to better understand the likely fate of hydrocarbon constituents introduced to the Gulf during the DWH oil spill.

In summary, the microbial capacity to respond to and degrade hydrocarbons resulting from the DWH spill has been primarily studied in the water column to date, but neglected in sediments. Here we were able to show that responses in sediments were different at the taxonomic, functional and genomic levels using a combination of 16S rRNA gene sequencing and screening of shotgun metagenomic data for the hydrocarbon metabolic potentials. The finding of significant differences in hydrocarbon degradation gene abundances in samples that exceeded EPA-BM compared with those that did not, combined with laboratory experiments demonstrating the ability of indigenous sediment microbial communities to degrade components in the oil, is compelling evidence that surface sediment-associated microbes can account for rapid depletion of some of the missing oil (at least short linear alkanes and simple aromatics). As discussed above, several lines of evidence suggested that PAHs may have persisted in sediments, which has ecological consequences that have only begun to be determined (for example, negative impacts on meiofauna diversity (Montagna *et al.*, 2013)). Therefore, our findings regarding the sediment microbial community response to accidental release of oil into the Gulf ecosystem should be considered in future scenarios describing the fate of the oil that was unaccounted for from the spill.
