# Supplementary material for: Metagenomics reveals sediment microbial community response to Deepwater Horizon oil spill
Source: ISME J. 2014 Jan 23;8(7):1464–75. doi: 10.1038/ismej.2013.254 (PMC4069396; doi:10.1038/ismej.2013.254)
Supplement: Supplementary Information [file ismej2013254x1.pdf]

Supplementary Material

Supplementary Tables.

Supplementary Table 1. Sediment metadata and chemistry.

Sample ID	Abbreviated Sample ID	Date collected	Latitude	Longitude	Distance from the wellhead (km)	Dissolved phosphate-P (PO_4-P) (µg/L)	Dissolved Inorganic Nitrogen (DIN) (µg/L)	Total Ammonia/Ammonium-N (TAN) (µg/L)	Dissolved Nitrate-N (NO_3-N) (µg/L)	Total Nitrogen (% dry weight)	Total Carbon (% dry weight)	Total Sulfur (% dry weight)	TPH (µg/kg)	Total Aromatics (µg/kg)	Total Alkanes (µg/kg)	Total Branched Alkanes (µg/kg)	Total Cyclic Alkanes (µg/kg)	Total Other Hydrocarbons (µg/kg)	Total PAH (µg/kg)
SD-20100916-GY01-FFC1-BC-001	BP001	9/16/10	28.059440	-90.248216	199.0	49	442	221	221	0.17	2.47	0.47	1162.57	61.79	66.68	387.44	642.99	3.67	0.00
SU-20100919-GY-FFC7-BC-007	BP007	9/19/10	27.733040	-89.976970	193.5	106	413	319	94	0.22	5.24	0.38	950.25	7.20	284.15	381.32	242.74	17.61	17.23
SD-20100919-GY-FFC4-BC-011	BP011	9/19/10	27.460420	-89.779460	198.6	79	421	64	357	0.16	3.81	0.59	778.98	37.30	27.33	246.43	467.92	0.00	0.00
SD-20100920-GY-FFMT6-BC-015	BP015	9/20/10	26.999740	-87.996710	196.7	52	230	77	153	0.06	6.57	0.18	1423.91	33.62	47.31	269.96	1073.02	0.00	0.00
SE-20100920-GY-FFMT5-BC-019	BP019	9/20/10	27.336320	-88.659340	158.6	0	0	0	0	0.10	1.60	0.02	324.57	13.63	100.78	111.94	92.82	4.12	1.29
SE-20100921-GY-FFMT4-BC-023	BP023	9/21/10	27.828320	-89.164780	128.0	68	445	246	199	0.13	3.44	0.36	2289.35	30.70	115.66	93.31	2049.68	0.00	0.00
SE-20100921-GY-FFMT3-BC-027	BP027	9/21/10	28.218674	-89.491710	124.3	74	669	450	218	0.19	2.45	0.50	200.77	3.61	19.44	104.99	72.73	0.00	0.00
SE-20100922-GY-LBNL3-BC-031	BP031	9/22/10	28.705230	-88.401670	5.1	76	493	141	353	0.20	2.76	0.46	43166.15	5.21	18966.67	14960.93	1916.04	3401.03	3916.28
SE-20100926-GY-NF-010-BC-053	BP053	9/26/10	28.757164	-88.388669	3.0	208	3048	2976	73	0.21	3.23	0.14	10550.75	67.88	3977.82	2630.04	954.36	421.96	2498.68
SE-20100926-GY-NF-011-BC-057	BP057	9/26/10	28.765306	-88.366883	3.0	283	5150	4403	747	0.19	3.26	0.25	17231.87	2721.86	4672.54	5039.96	4434.20	69.26	294.06
SE-20100926-GY-NF-012-BC-058	BP058	9/26/10	28.757853	-88.344461	3.0	184	2375	1921	454	0.20	3.74	0.21	1646.10	253.09	440.13	481.42	417.53	25.19	28.74
SE-20100927-GY-NF013-BC-062	BP062	9/27/10	28.738786	-88.351408	1.4	148	571	494	77	0.18	2.45	0.50	1370.66	24.97	533.61	459.79	232.71	6.78	112.81
SE-20100927-GY-NF014-BC-066	BP066	9/27/10	28.719603	-87.292068	104.7	108	635	476	159	0.18	2.58	0.31	4315.24	18.08	2053.40	762.23	1373.97	6.16	101.40
SE-20100927-GY-ALTNF015-BC-070	BP070	9/27/10	28.709925	-88.366436	3.2	84	436	335	102	0.27	3.82	0.62	3802.30	27.34	1592.57	904.00	293.21	118.63	866.54
SE-20100927-GY-LBNL17-BC-074	BP074	9/27/10	28.699537	-88.380993	4.6	128	1604	1091	513	0.20	3.69	0.38	65643.67	22.10	25237.24	22186.05	2915.65	6206.72	9075.92
SE-20100928-GY-NF008-BC-093	BP093	9/28/10	28.720005	-88.388440	3.1	148	2456	2301	155	0.18	3.84	0.24	1494.88	16.19	168.64	160.96	1128.78	6.46	13.84
SE-20100928-GY-NF-009-BC-097	BP097	9/28/10	28.738219	-88.397370	3.1	205	1152	1020	131	0.21	3.16	0.59	3383.13	35.05	1410.36	1285.46	448.26	98.99	105.00
SE-20100928-GY-LBNL05-BC-101	BP101	9/28/10	28.672508	-88.435906	10.1	97	3757	2105	1653	0.16	3.18	0.19	1714.56	14.75	95.71	118.75	1467.93	14.78	2.64
SE-20101001-GY-LBNL1-BC-120	BP120	10/1/10	28.731900	-88.376640	1.3	78	632	623	9	0.14	2.14	0.13	2287.67	17.85	454.92	188.38	1582.17	21.84	22.52
SE-20101001-GY-ALTNF001-BC-139	BP139	10/1/10	28.734720	-88.370510	0.7	120	3705	2501	1204	0.15	3.54	0.59	21721.36	39.64	3262.47	11760.25	2853.58	3704.72	100.70
SE-20101001-GY-NF006MOD-BC-143	BP143	10/1/10	28.745110	-88.359140	0.9	104	3450	3198	252	0.22	4.84	0.57	13188.94	92.87	5558.24	5167.49	316.09	814.62	1239.63
SE-20101002-GY-FF005-BC-147	BP147	10/2/10	28.806810	-88.561090	20.5	142	1576	814	762	0.16	2.72	0.16	11103.35	0.00	4933.85	3702.40	1781.61	414.52	270.97
SE-20101002-GY-FF010-BC-151	BP151	10/2/10	28.667880	-88.429990	10.1	126	719	421	298	0.18	2.70	0.37	17229.54	0.00	3715.58	8564.00	1238.09	2426.54	1285.33
SE-20101002-GY-LBNL7-BC-155	BP155	10/2/10	28.639060	-88.471320	15.1	140	3986	3709	277	0.19	3.78	0.20	1993.46	0.00	1517.90	196.86	157.81	32.20	88.70
SE-20101003-GY-FF004-BC-174	BP174	10/3/10	28.834110	-88.650020	29.7	102	542	436	106	0.22	3.17	0.35	464.03	9.08	158.49	172.80	116.74	0.00	6.92
SE-20101008-GY-D015S-BC-178	BP178	10/8/10	28.297260	-88.636490	55.8	77	422	287	134	0.22	5.35	0.68	991.92	0.00	610.51	27.21	23.06	331.14	0.00
SE-20101008-GY-ALTFF012-BC-182	BP182	10/8/10	28.297471	-88.636310	55.7	139	614	513	101	0.17	3.64	0.44	18609.18	0.00	1515.35	12026.57	1587.76	2775.60	703.89
SE-20101008-GY-LBNL11-BC-186	BP186	10/8/10	28.345180	-88.778520	59.5	94	1487	1260	227	0.14	2.66	0.19	1319.27	17.83	244.16	600.71	403.76	26.50	26.31
SE-20101008-GY-FF011-BC-205	BP205	10/8/10	28.511000	-88.529940	30.0	130	615	119	496	0.17	2.47	0.20	203.47	5.48	33.65	97.42	62.89	4.03	0.00
SE-20101009-GY-D017S-BC-209	BP209	10/9/10	28.473360	-88.478320	31.5	79	642	187	455	0.20	2.88	0.32	18.63	0.00	3.96	10.31	4.37	0.00	0.00
SE-20101009-GY-D014S-BC-213	BP213	10/9/10	28.565410	-88.448070	20.9	113	811	343	468	0.17	3.24	0.45	11278.97	0.00	1340.84	6479.73	871.36	1807.05	779.99
SE-20101009-GY-D019S-BC-217	BP217	10/9/10	28.672710	-88.347467	7.6	120	264	95	169	0.17	2.38	0.37	4116.66	0.00	402.98	2698.38	302.01	476.92	236.36
SE-20101009-GY-FF003-BC-221	BP221	10/9/10	28.873950	-88.756890	41.0	120	1188	607	581	0.21	2.92	0.35	1281.95	40.09	376.41	249.09	563.11	11.47	41.78
SE-20101009-GY-ALTFF002-BC-225	BP225	10/9/10	28.939910	-88.893090	56.0	402	3558	2344	1214	0.20	2.31	0.20	3633.75	157.77	430.61	369.72	2626.08	20.09	29.48
SE-20101009-GY-FF001-BC-229	BP229	10/9/10	28.968860	-89.029940	69.6	330	2051	1778	274	0.24	2.38	0.56	420.13	0.00	9.17	344.80	66.16	0.00	0.00
SE-20101010-GY-D062S-BC-233	BP233	10/10/10	28.265650	-88.923320	75.7	82	407	223	184	0.36	6.60	0.80	400.82	4.04	128.17	21.66	240.59	1.04	5.32
SE-20101010-GY-FF013-BC-237	BP237	10/10/10	28.204850	-89.056010	89.9	134	452	319	133	0.16	2.16	0.34	656.83	8.32	94.38	66.64	483.59	3.89	0.00
SE-20101010-GY-D053S-BC-241	BP241	10/10/10	27.651170	-89.282010	150.6	114	466	222	244	0.14	5.27	0.32	6887.47	0.00	494.71	4766.35	569.20	863.31	193.90
SE-20101011-GY-D064S-BC-245	BP245	10/11/10	27.359255	-90.568790	265.1	75	437	235	202	0.21	5.40	0.58	14530.28	0.00	962.41	9827.34	1516.45	1805.73	418.35
SE-20101012-GY-D007S-BC-249	BP249	10/12/10	28.086580	-88.516990	74.0	88	436	203	233	0.17	4.35	0.42	532.82	7.15	83.81	185.32	254.10	1.11	1.33
SE-20101012-GY-D008S-BC-253	BP253	10/12/10	27.887420	-88.626810	98.1	53	252	117	135	0.14	4.30	0.07	248.16	4.27	24.43	13.19	205.54	0.00	0.73
SE-20101012-GY-D013S-BC-257	BP257	10/12/10	27.654381	-88.637922	123.5	94	463	264	199	0.18	6.77	0.11	8803.26	0.00	634.42	5994.83	764.27	1201.19	208.55
SE-20101012-GY-D003S-BC-261	BP261	10/12/10	28.116710	-88.071790	74.9	46	384	125	259	0.13	4.28	0.44	13933.13	0.00	647.32	9794.62	1354.81	1609.43	526.94
SE-20101012-GY-D006S-BC-265	BP265	10/12/10	28.343090	-88.139740	49.2	74	351	117	234	0.18	5.02	0.45	1698.49	1.81	25.38	40.21	1621.23	4.71	5.14
SE-20101013-GY-D055S-BC-269	BP269	10/13/10	28.415710	-88.725590	50.3	100	455	214	241	0.15	1.71	0.02	193.84	2.64	49.88	77.57	57.66	4.43	1.67
SE-20101013-GY-FFMT2-BC-273	BP273	10/13/10	28.447920	-89.671880	131.6	89	475	379	97	0.21	2.92	0.44	158.48	0.00	0.08	154.82	3.58	0.00	0.00
SE-20101017-GY-D031S-BC-278	BP278	10/17/10	28.715039	-88.358730	2.7	113	5598	4541	1057	0.22	4.77	1.00	39831.08	24.90	19264.44	11033.34	1620.99	1846.18	6041.22
SE-20101017-GY-D034S-BC-296	BP296	10/17/10	28.734820	-88.362210	0.5	77	994	871	123	0.16	2.44	0.27	311.76	0.00	0.00	76.64	235.12	0.00	0.00
SE-20101017-GY-D040S-BC-315	BP315	10/17/10	28.742300	-88.362202	0.5	164	4135	3484	651	0.12	4.26	0.32	49367.23	105.84	29338.63	10791.74	1540.88	2341.40	5248.74
SE-20101017-GY-D038SW-BC-331	BP331	10/17/10	28.740480	-88.368060	0.3	93	703	430	273	0.12	5.06	0.15	22107.69	0.00	11277.95	6611.10	554.49	1083.20	2580.95
SE-20101017-GY-D042S-BC-350	BP350	10/17/10	28.742530	-88.370510	0.6	140	3584	2468	1116	0.17	6.17	0.35	31861.75	84.41	17826.25	7574.57	1719.50	1142.24	3514.77
SE-20101017-GY-D044S-BC-366	BP366	10/17/10	28.744920	-88.374240	1.1	127	3567	3450	117	0.20	3.55	0.30	5231.60	0.00	3152.08	1393.91	477.05	0.00	208.56
SE-20101018-GY-D002S-BC-382	BP382	10/18/10	28.557090	-87.639269	73.7	47	188	110	79	0.16	4.60	0.42	9328.92	11.22	575.35	6309.48	795.99	1108.35	528.53
SE-20101018-GY-D004S-BC-386	BP386	10/18/10	28.582635	-87.885728	49.9	69	486	276	209	0.16	4.20	0.43	63.69	0.00	15.47	41.80	6.42	0.00	0.00
SE-20101018-GY-D010S-BC-390	BP390	10/18/10	28.570090	-87.023350	132.3	144	887	512	375	0.30	5.03	0.63	25724.23	0.00	2161.89	16004.26	1975.40	4050.42	1532.27
SE-20101018-GY-D012S-BC-394	BP394	10/18/10	28.672440	-88.233930	14.8	110	1878	652	1226	0.17	3.82	0.31	278.12	6.39	62.05	80.31	122.78	4.00	2.58
SE-20101018-GY-D021S-BC-398	BP398	10/18/10	28.703040	-88.360950	4.0	108	661	352	309	0.16	2.25	0.45	31709.11	0.00	3307.64	18177.23	2209.22	5850.33	2164.68
SE-20101019-GY-D068S-BC-402	BP402	10/19/10	28.710900	-88.748320	37.5	138	895	695	200	0.19	2.29	0.38	1202.19	26.97	447.52	336.21	277.08	56.77	57.64
SE-20101019-GY-LBNL4-BC-425	BP425	10/19/10	28.668080	-88.417295	9.3	125	1130	876	253	0.20	2.88	0.76	34720.26	0.00	2752.64	23086.34	2303.46	5123.61	1454.20
SE-20101019-GY-LBNL8-BC-444	BP444	10/19/10	28.575210	-88.537840	24.8	86	570	305	265	0.26	2.70	0.33	25577.56	0.00	2961.45	15468.32	1533.49	3491.35	2122.94
SE-20101019-GY-LBNL9-BC-463	BP463	10/19/10	28.514140	-88.600570	33.9	83	489	375	113	0.24	3.07	0.54	33174.53	0.00	5734.97	18546.56	2013.68	4626.96	2252.37
SE-20101019-GY-LBNL13-BC-482	BP482	10/19/10	28.447050	-88.759340	50.3	102	787	434	353	0.17	2.11	0.56	23323.55	0.00	3859.32	10345.98	2984.71	4396.95	1736.59
SE-20101019-GY-LBNL10-BC-501	BP501	10/19/10	28.415570	-88.704270	48.8	86	384	272	112	0.21	1.97	0.25	22408.89	0.00	4120.89	12002.37	1951.28	3108.19	1226.16
SE-20101020-GY-LBNL12-BC-520	BP520	10/20/10	28.324660	-88.939660	72.6	81	558	461	97	0.19	2.13	0.68	184.60	4.97	68.65	44.89	53.70	0.00	12.39

Metagenomic sequencing was carried out on samples highlighted in blue and red. Samples that exceeded the EPA-BM are highlighted in red.

Supplementary Figures.

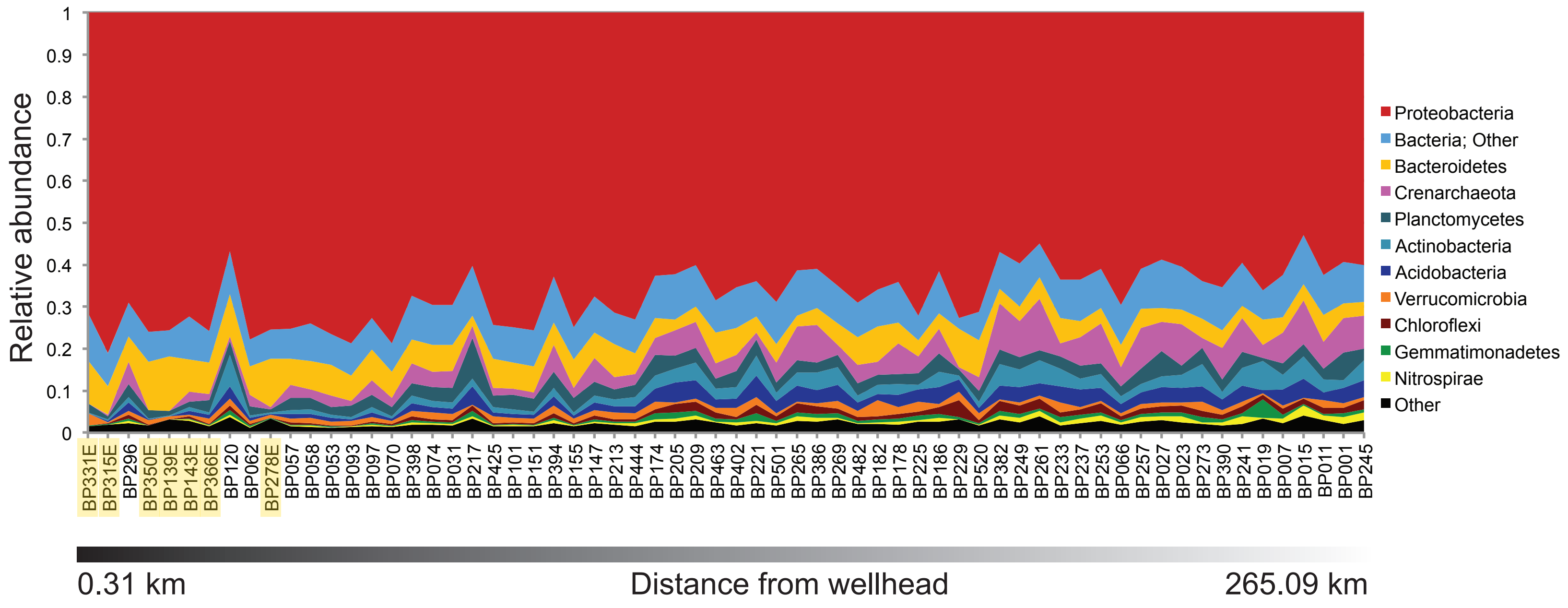

Supplementary Figure 1. Microbial community structure as determined by iTag sequencing of 16S rRNA genes. Samples that exceeded the EPA-BM are denoted by “E” following the sample name and are highlighted.

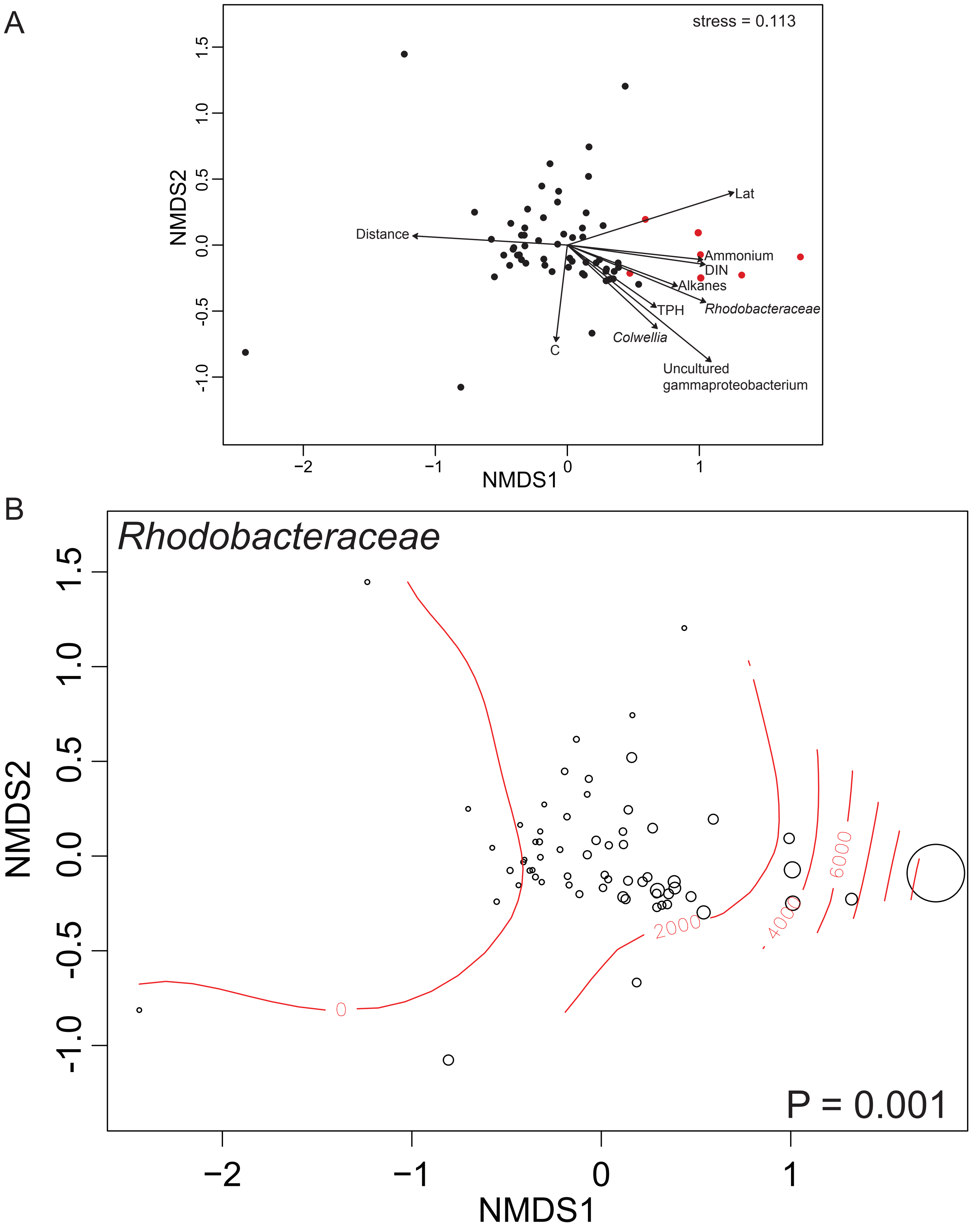

Supplementary figure 2. A) ordination of 16S rRNA gene iTag data. B) The same ordination showing *Rhodobacteraceae* abundance (bubble size indicates abundance).

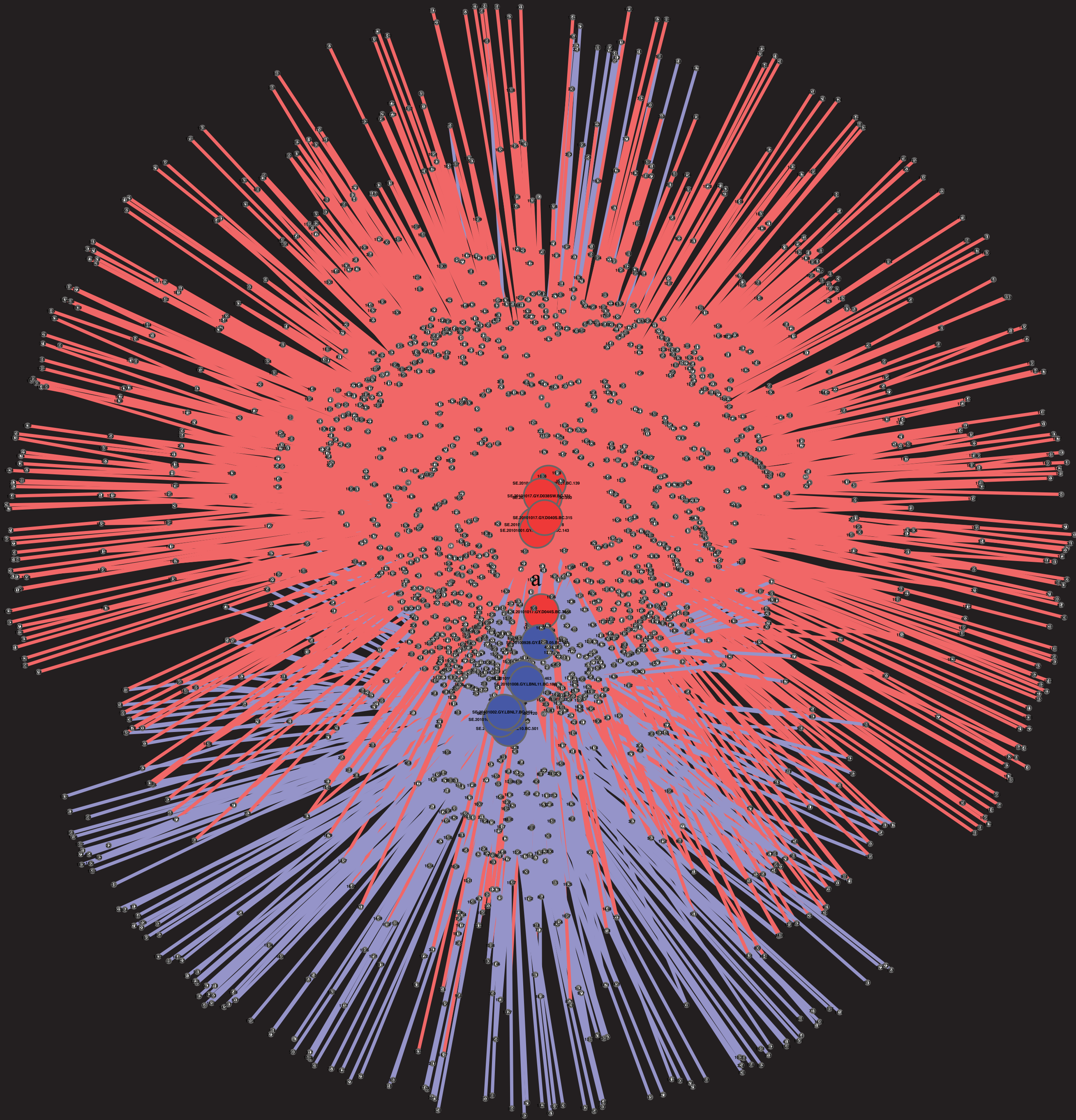

Supplementary Figure 3. Network analysis of metagenome data annotated with MG-RAST Hierarchical Classification. Samples that exceeded the EPA-BM are shown in red. Samples that did not exceed the EPA-BM are shown in blue. Samples cluster based on their shared genes, with those sharing more genes being closer together.

Supplementary Figures.

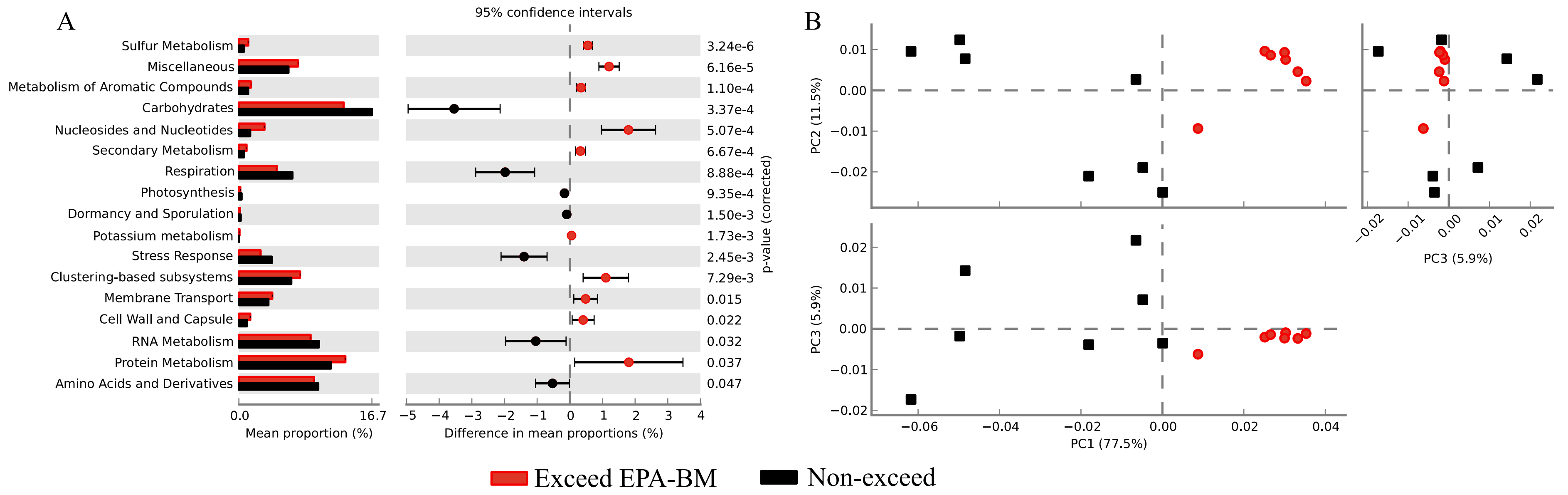

Supplementary Figure 4. Statistical analysis of metagenome data annotated with MG-RAST. A) Comparison of functional categories of MG-RAST gene annotations in samples that exceed EPA-BM compared to those that did not. B) Ordination of gene functions.

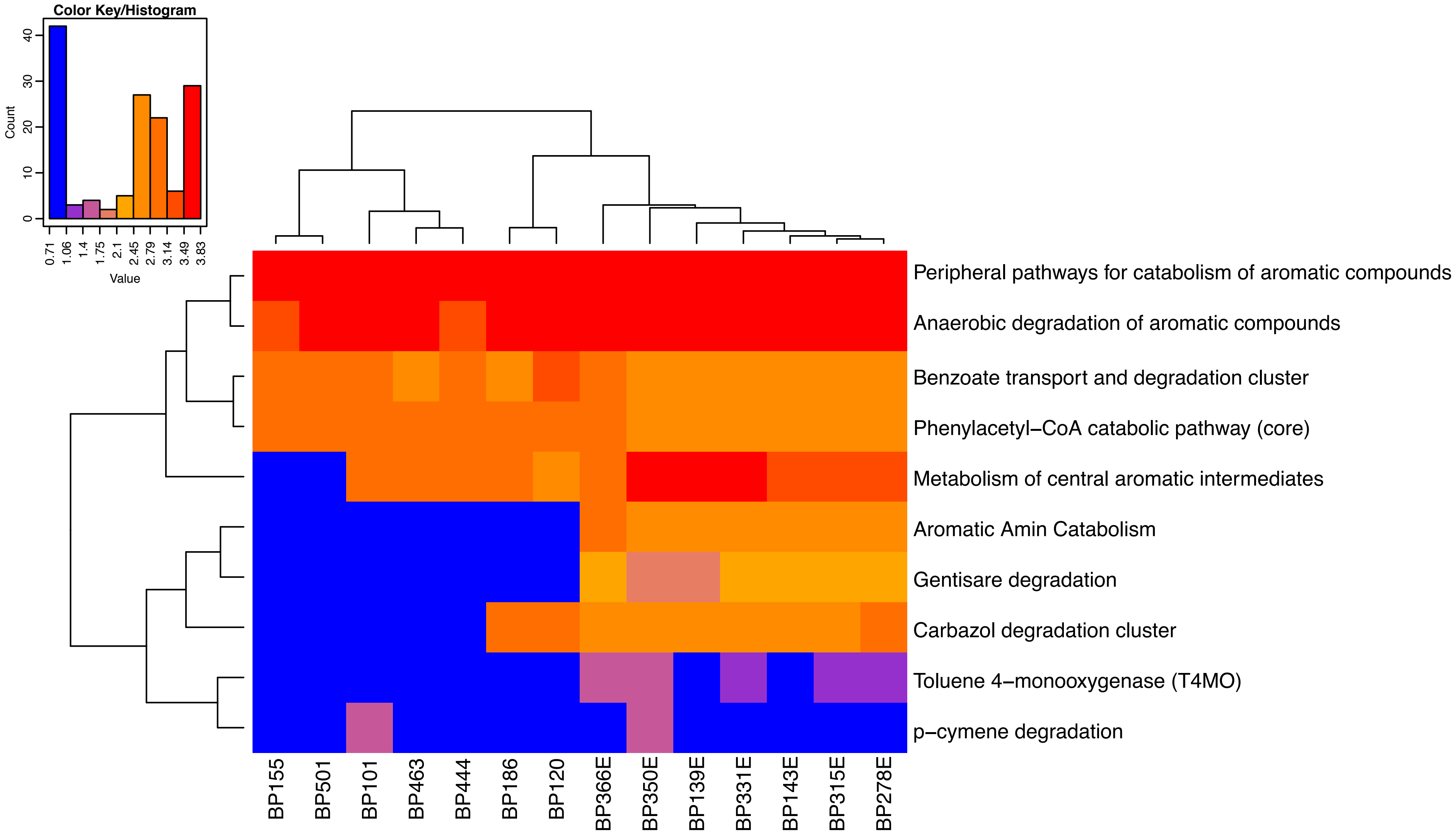

Supplementary Figure 5. Heatmap of unassembled metagenomic data annotated using MG-RAST hierarchical clustering.

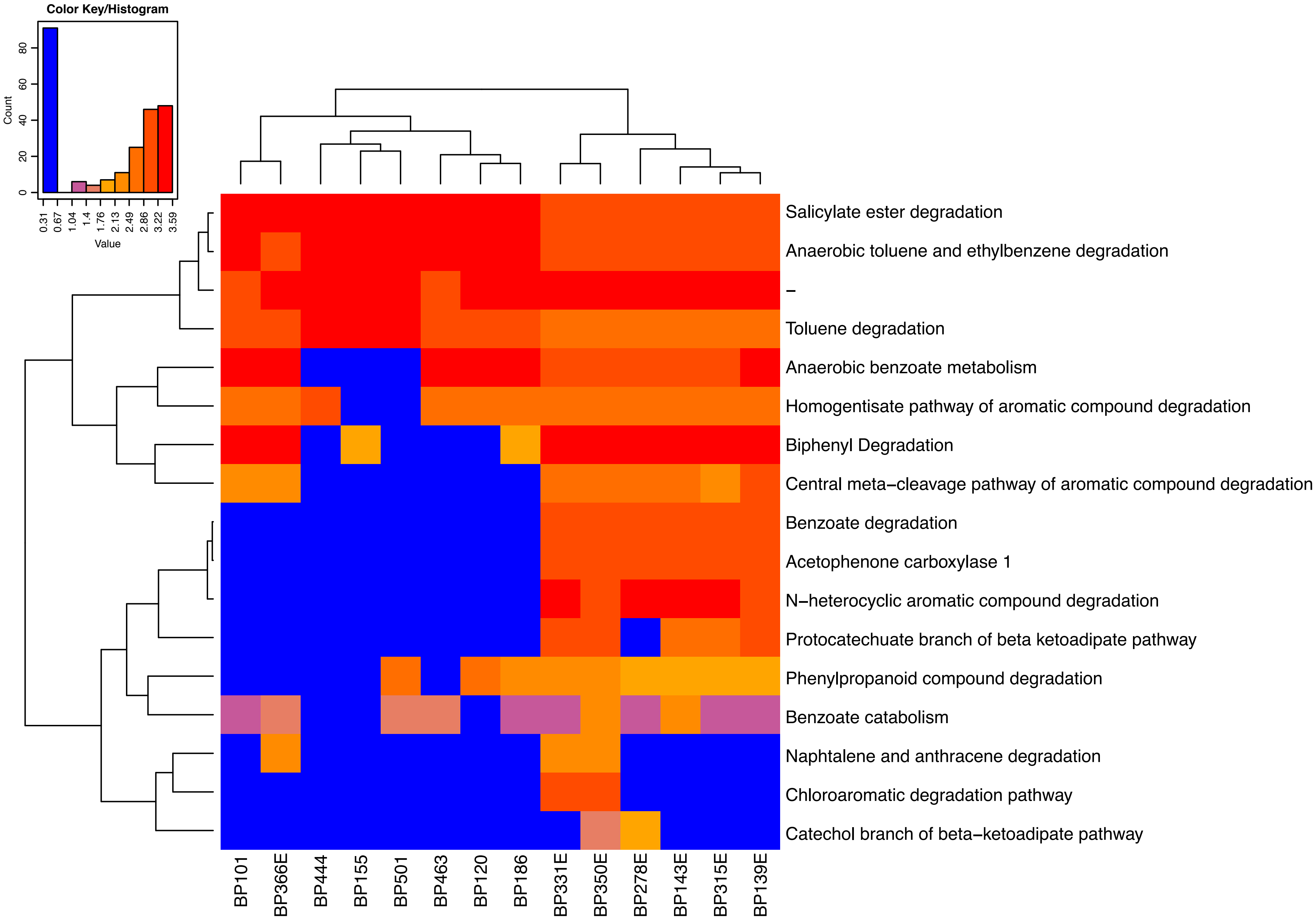

Supplementary Figure 6. Heatmap of unassembled metagenomic data annotated using MG-RAST hierarchical clustering.

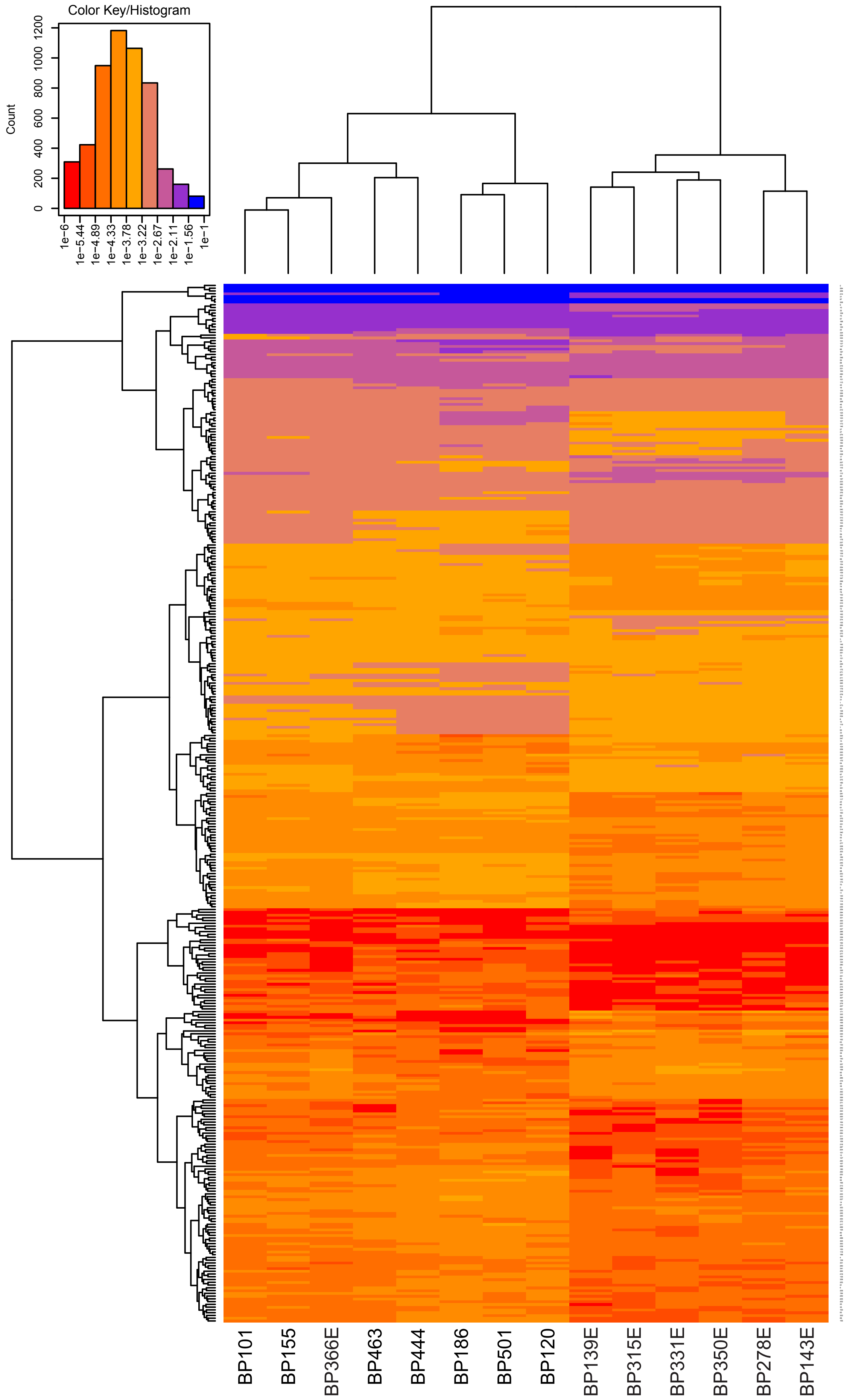

Supplementary Figure 7. Raw, unassembled metagenomic reads compared to a database of genes involved in hydrocarbon degradation.

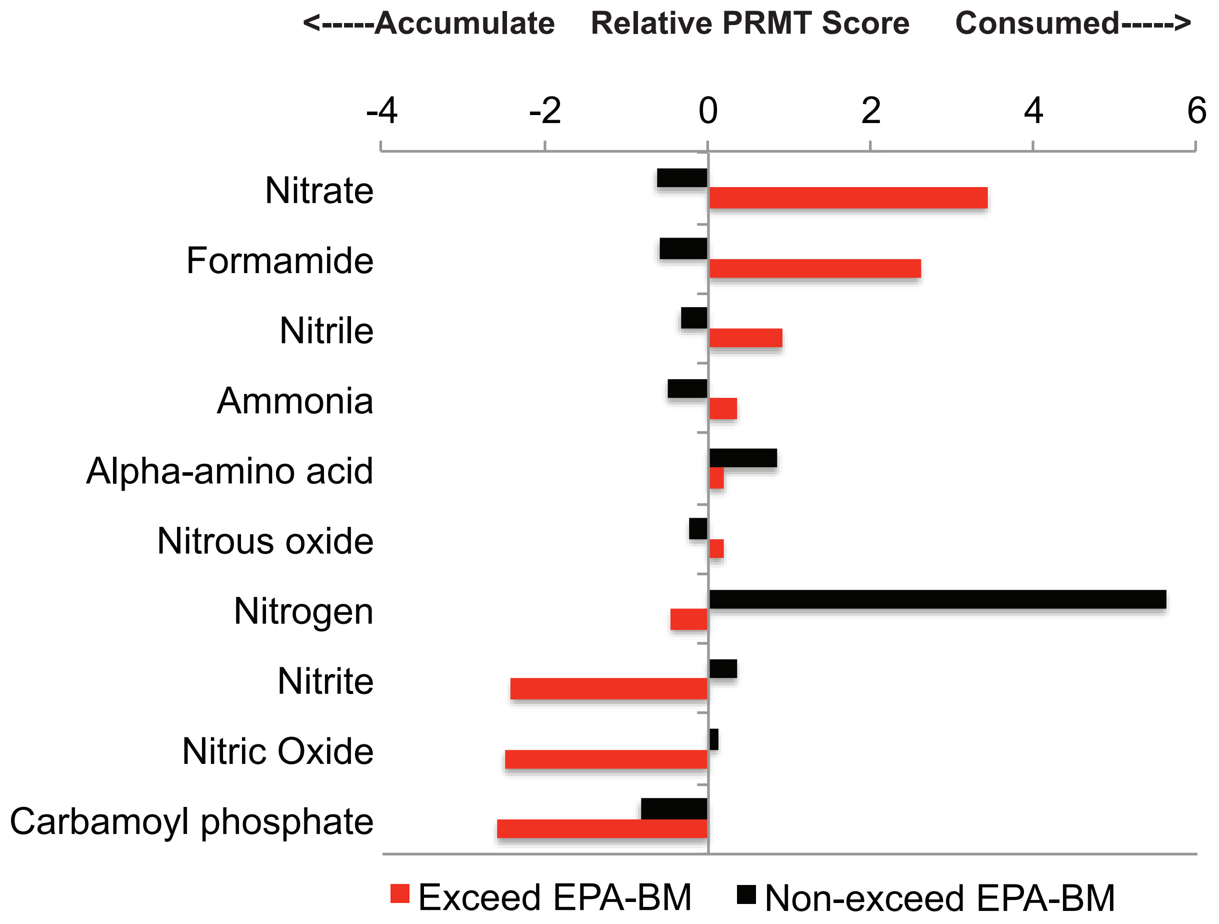

Supplementary Figure 8. PRMT analysis of unassembled metagenomic reads annotated with MG-RAST SEED.

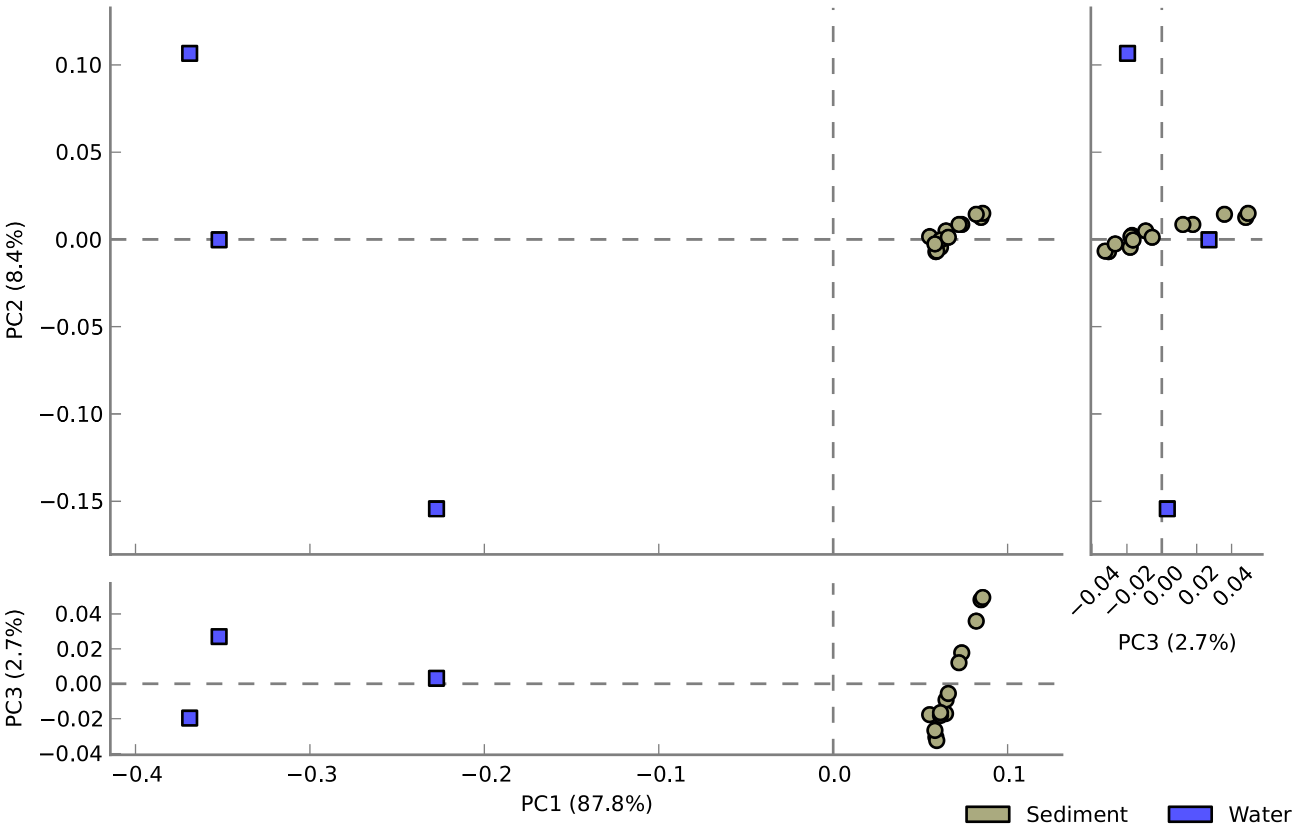

Supplementary Figure 9. Comparison of metagenomic sequence data annotated by comparing raw, unassembled metagenomic reads to a database of genes involved in hydrocarbon degradation. Sediment samples are from this study. Water column samples are from the deep-sea plume dataset presented in Mason OU, Hazen TC, Borglin S, Chain PSG, Dubinsky EA, Fortney JL, et al. (2012). Metagenome, metatranscriptome and single-cell sequencing reveal microbial response to Deepwater Horizon oil spill. The ISME journal 6:1715–27.
